# Immune Dysregulation and Infectious Complications in MPN Patients Treated With JAK Inhibitors

**DOI:** 10.3389/fimmu.2021.750346

**Published:** 2021-11-19

**Authors:** Daniele Cattaneo, Alessandra Iurlo

**Affiliations:** ^1^ Hematology Division, Foundation Istituto di Ricovero e Cura a Carattere Scientifico (IRCCS) Ca’ Granda Ospedale Maggiore Policlinico, Milan, Italy; ^2^ Department of Oncology and Hemato-Oncology, University of Milan, Milan, Italy

**Keywords:** myeloproliferative neoplasms, JAK inhibitors, ruxolitinib, infections, immune system

## Abstract

*BCR-ABL1*-negative myeloproliferative neoplasms are burdened by a reduced life expectancy mostly due to an increased risk of thrombo-hemorrhagic events, fibrotic progression/leukemic evolution, and infectious complications. In these clonal myeloid malignancies, *JAK2*V617F is the main driver mutation, leading to an aberrant activation of the Janus kinase-signal transducer and activator of transcription (JAK-STAT) signaling pathway. Therefore, its inhibition represents an attractive therapeutic strategy for these disorders. Several JAK inhibitors have entered clinical trials, including ruxolitinib, the first JAK1/2 inhibitor to become commercially available for the treatment of myelofibrosis and polycythemia vera. Due to interference with the JAK-STAT pathway, JAK inhibitors affect several components of the innate and adaptive immune systems such as dendritic cells, natural killer cells, T helper cells, and regulatory T cells. Therefore, even though the clinical use of these drugs in MPN patients has led to a dramatic improvement of symptoms control, organ involvement, and quality of life, JAK inhibitors–related loss of function in JAK-STAT signaling pathway can be a cause of different adverse events, including those related to a condition of immune suppression or deficiency. This review article will provide a comprehensive overview of the current knowledge on JAK inhibitors’ effects on immune cells as well as their clinical consequences, particularly with regards to infectious complications.

## 1 Introduction

The *BCR-ABL1*-negative myeloproliferative neoplasms (MPNs) are a heterogenous group of clonal disorders of the hematopoietic stem cell, mainly characterized by hyperproliferative bone marrow with varying degrees of reticulin/collagen fibrosis, extramedullary hematopoiesis, abnormal peripheral blood counts, and constitutional symptoms. They include polycythemia vera (PV), essential thrombocythemia (ET), and myelofibrosis (MF). The latter may present as a primary disorder (PMF) or evolve from another pre-existing *BCR-ABL1*-negative MPN, such as PV or ET, globally identified as secondary MF (SMF) (1).

Somatic mutations in MPNs are classified into “driver” and “other” mutations; the former include *JAK2*, *CALR*, and *MPL*; and the latter, *ASXL1*, *EZH2*, *IDH1/2*, *SRSF2* and *U2AF1*, among others (2–4). It is generally believed that driver mutations are essential for MPN phenotype, whereas the “other” mutations might contribute to fibrotic progression and leukemic evolution (5, 6).

*JAK2*V617F is the main driver mutation in MPNs, leading to an aberrant activation of the Janus kinase-signal transducer and activator of transcription (JAK-STAT) signaling pathway. Therefore, its inhibition represents an attractive therapeutic strategy for these disorders.

Numerous JAK inhibitors have entered clinical trials, including ruxolitinib, the first JAK1/2 inhibitor to become commercially available for the treatment of MPNs. Indeed, it was initially approved in both the US and Europe for the treatment of splenomegaly and/or constitutional symptoms in MF patients (7, 8); subsequently, it was also licensed for PV subjects with an inadequate response to or an unacceptable toxicity from hydroxyurea (HU) (9, 10).

Despite its efficacy, ruxolitinib-related loss of function in JAK-STAT signaling pathway can be a cause of different adverse events (AEs), including those related to a condition of immune suppression or deficiency. Accordingly, the increased risk of infections already inherent to even untreated MPNs is further augmented due to the immunomodulatory and immuno-suppressive effects of JAK inhibitors.

This review article will provide a comprehensive overview of the current knowledge on JAK inhibitors’ effects on immune cells, as well as their clinical consequences particularly with regards to infectious complications.

## 2 Immunosuppressive Activity of JAK Inhibitors

The JAK-STAT pathway—based on four non-receptor protein tyrosine kinases, JAK1, JAK2, JAK3, and Tyk2, and seven STAT proteins—regulates proliferation, differentiation, and survival of a variety of cells and is crucially relevant for hematopoiesis as well as for immune cell development and function (11–14). As an example, JAK1 is involved in type I IFNs, IFN-γ, IL-2, IL-7, IL-15, and IL-21 signaling; it also cooperates with Tyk2 for type I IFN and with JAK2 for IFN-γ signal transduction (15–17). JAK2 is activated by several cytokines and growth factors including erythropoietin, thrombopoietin, IL-3, IL-6, G-CSF, and IFN-γ (18, 19), while JAK3 is associated with the Υc chain family of cytokines (20) and Tyk2 is triggered by cytokines such as type I IFNs, IL-6, IL-10, and the IL-12 and IL-23 families (21).

Due to interference with the JAK-STAT pathway, JAK inhibitors affect several components of the innate and adaptive immune systems such as dendritic cells (DCs), natural killer cells (NKs), T helper cells, and regulatory T cells (Tregs), thus resulting in a significant immunosuppressive activity (**Table 1**) (27).

**Table 1:** Immune system components and JAK inhibitors’ effects.

	Physiological function	JAK inhibitors’ effects	References
Dendritic cells (DCs)	Antigen-presenting/phagocytic cells responsible for presenting antigens to T lymphocytes	Ruxolitinib: inhibition of DCs development, activation, and migration Momelotinib: reduced CD80/CD86 expression involved in T-cell activation, expansion, and differentiation	(22) (23)
Natural Killer cells (NKs)	Cytotoxic lymphocytes that play a critical role in antiviral and antitumor responses	Ruxolitinib: impaired maturation as reflected by an increased ratio in immature to mature NKs; reduced capacity to form lytic synapses with NK target cells	(24)
CD4+ T cells	Heterogeneous cell population involved in adaptive immunity, inflammatory response, and protection against a wide range of both intracellular and extracellular pathogens	Ruxolitinib: severe, long-lasting reduction of circulating regulatory T cells not reversible upon drug reduction or withdrawal Ruxolitinib/fedratinib: “silence” CD4+ T cells and decrease cytokine secretion; polarize the immune profile toward a “Th17 type” response	(25) (26)

### 2.1 Dendritic Cells

Dendritic cells are crucial antigen-presenting and phagocytic cells responsible for presenting antigens to T lymphocytes, initiating therefore the process of adaptive immunity (28, 29). The pivotal study by Heine et al. on ruxolitinib-induced alterations of DCs function laid the preliminary bases for understanding the increased infection rate recorded in MPN patients treated with this JAK inhibitor as well as its anti-inflammatory and immunomodulating activity. The *in vitro* development of human monocyte-derived DCs was almost completely blocked, and DCs activation was inhibited as shown by decreased IL-12 production. Dendritic cell migration both *in vitro* and *in vivo* in mice was also reduced, resulting in impaired T-cell activation (22).

The impact of ruxolitinib on DCs migration and the identification of target molecules mediating this effect were further investigated by means of an *ex vivo* assay. Dendritic cell migration turned out to be heavily depressed *via* interference with Rho-associated coiled-coil kinase (ROCK) that controls non-muscle myosin activity regulating reorganization and contraction of cellular actin–myosin filaments. This DCs loss of mobility may lead to a reduced T cell activation in draining lymph nodes and might explain the increased number of these proinflammatory blood cells in ruxolitinib-treated MPN patients (30).

Similar to ruxolitinib, momelotinib impacts on DCs’ functions. In a mouse model of atopic dermatitis, besides inhibiting mRNA expression of IL-4, IL-5, IFN-γ and STAT1, STAT3 and STAT5 phosphorylation in topically treated skin lesions, momelotinib reduced in activated DCs *in vitro* the expression of the co-stimulatory molecules CD80 and CD86 involved in T-cell activation, expansion, and differentiation (23). On the contrary, in a more recent paper, another JAK inhibitor, pacritinib, exhibited only a mild suppressive effect on DCs. Pacritinib, at concentrations reflecting patients’ plasma levels, reduced IL-12 secretion, whereas IL-6 and TNF-α levels were unchanged, thus producing an immunosuppressive effect on DCs significantly less pronounced than that of ruxolitinib (31).

### 2.2 Natural Killer Cells

Natural Killer cells are cytotoxic lymphocytes of the innate immune system that play a critical role in antiviral and antitumor responses. The coordinated action of multiple cytokines is crucial for NKs development and maturation, and many of these cytokines such as IL-2, IL-7, IL-12, IL-15, IL-21, IL-27, and IFNs signal *via* the JAK-STAT pathway (32). The effects of JAK inhibition on human NKs was extensively investigated in a study comparing 28 MPN patients with or without ruxolitinib treatment and 24 healthy subjects. In ruxolitinib-treated subjects, a decrease in NKs number associated with clinically relevant infections mostly of viral origin was recorded likely due to impaired maturation as reflected by an increased ratio in immature to mature NKs. Also, the endogenous defect in NKs function of MPN patients was further worsened by ruxolitinib. These *in vivo* findings were supported by *in vitro* data showing that the cytokine-mediated NKs activation was inhibited by ruxolitinib as suggested by a reduced expression of NKs activation markers such as CD16, CD69, NKG2D, NKp46, and granzyme B. A diminished killing activity was also reported due to an impaired capacity to form lytic synapses with NK target cells. The *in vitro* ruxolitinib effects on NKs’ function were restored upon drug removal, indicating reversibility of this action (24).

The impact of ruxolitinib and fedratinib, a JAK2-specific inhibitor, on NKs activation and function were then compared *in vitro* using γc cytokines and human DCs subtypes. While ruxolitinib completely blocked IL-2, IL-15, and DC-mediated STAT5 phosphorylation, along with the capacity of NKs to secrete IFNγ and lyse NK-sensitive targets, fedratinib inhibited only soluble IL-15-mediated STAT5 phosphorylation, which Langerhans-type DCs, presenting membrane-bound IL-15 *in trans*, could salvage, demonstrating that a selective JAK2 inhibitor better preserves NKs activity (33).

Ruxolitinib effect on NKs was evaluated also in the context of host immune response against gene therapy viral vectors by means of a co-culture system with human NKs line, macrophages, and airway epithelial cells. The increased IFN-γ cytokine expression induced by NKs co-cultured with helper-dependent adenoviral (HD-Ad) vector-activated macrophages as well as the kill of HD-Ad vector-transduced bronchial epithelial cells by activated NKs were both significantly reduced by ruxolitinib due to a block of IL-12 and IL-15 production (34).

### 2.3 CD4+ T Cells

CD4+ T cells, a heterogeneous cell population differentiating into multiple effector subsets such as T-helper and Tregs, play a central role in adaptive immunity, mediate inflammatory response, and protect against a wide range of both intracellular and extracellular pathogens by releasing cytokines and chemokines that induce and/or recruit target cells (35, 36). The effect of JAK inhibition on CD4+ T cells was firstly investigated by Massa et al. on 18 MF patients: the administration of ruxolitinib resulted in a severe, long-lasting reduction of circulating Tregs not reversible upon drug reduction or withdrawal. It was suggested that decreased levels of Tregs by disrupting the immune response might explain the increased frequency of infections, such as tuberculosis, Herpes zoster, and pneumonia of viral origin reported in ruxolitinib-treated MF patients (25). Similar results were obtained in a study on nine MPN patients. After 3 weeks of ruxolitinib treatment, a decrease in total CD3+ cells, number of Tregs, Th1, and Th17 was observed; moreover, in T cells isolated from these patients, TNF-α, IL-5, IL-6, and IL-1B production was downregulated (37).

Frequency and function of CD4+ T cell subsets at baseline and during treatment with either ruxolitinib or fedratinib were further investigated in 50 MPN subjects. At baseline, Tregs in MPN patients were significantly lower than in 23 healthy controls, and all subgroups of Tregs as defined by CD45RA expression were further decreased by JAK inhibitors with no targeting of specific subpopulations. After 6 months of treatment in responsive subjects, a significant increase in Th17 cells compared to baseline and an expansion of “dual positive” IFNγ+/IL‐17+ cells were recorded, suggesting a polarization from a “Th1” to a “Th17” phenotype. A functional “silencing” of T helper cells both *in vivo* and *in vitro* and a significant decrease of pro‐inflammatory cytokines secretion by CD4+ T cells were also observed, thus representing a possible explanation for the increased rate of atypical infections reported in JAK inhibitors-treated subjects. It was suggested that JAK inhibitors may display a dual effect on number and function of CD4+ T cells, the early one being to “silence” CD4+ T cells and decrease cytokine secretion and the long-term one being to polarize the immune profile toward a “Th17 type” response (26).

The role of JAK inhibition specificity on T-cell proliferation and function was also investigated by evaluating the effects of ruxolitinib and momelotinib as JAK1/2 inhibitors and of BSK805 as selective JAK2 inhibitor. In T-cells derived from ruxolitinib-treated MPN patients, CD69 expression and proliferative capacity were almost abrogated in CD8+ and significantly impaired in CD4+ T cells, confirming that JAK1/2 inhibitors significantly decrease T-cell reactivity and proliferation *in vivo*. Ruxolitinib, together with momelotinib and BSK805, was also assessed *in vitro* on healthy donors T cells. In a mixed lymphocyte culture assay, while JAK1/2 inhibition significantly decreased T-cell reactivity, JAK2 specific blockage did not have any inhibitory effect, indicating that the T-cell function impairment is strictly dependent upon JAK1 inactivation (38).

## 3 JAK Inhibitors-Associated Infectious Complications in MPN Patients

Myeloproliferative neoplasms are burdened by a reduced life expectancy mostly due to an increased risk of thrombo-hemorrhagic events, fibrotic progression/leukemic evolution, and infections. Indeed, an intrinsic MPN propensity to infectious complications was firstly suggested by Swedish investigations performed before JAK inhibitors’ introduction. In a cohort of 9.285 MPN subjects, the 10-year probability of death from infections was 4.6% in PV, 2.5% in ET, and 10.4% in PMF compared to 2.3% in 35.769 matched controls (39). In a further study including 8.363 MPN cases and 32.405 controls, the hazard ratio (HR) of any infection was 2.0. According to MPN subtypes, the HR was 3.7 in PMF, and 1.7 in both PV and ET, with no significant difference between untreated patients and subjects treated during the years 2006–2013 with HU, IFN-α, or anagrelide. During the follow-up, however, the rate of infections raised in patients subsequently treated with ruxolitinib (40).

The issue that the inherent MPN susceptibility to infections might be increased by JAK inhibitors was addressed in clinical trials, retrospective series, case reports, and reviews (**Table 2**).

**Table 2:** JAK inhibitors-associated infectious complications.

	MPN subtype	References
Urinary tract infection	MF	(41–47)
Herpes zoster	MF PV	(41, 42, 48, 49) (9, 10, 50–53)
Herpes simplex reactivation	MF	(42)
Pneumonia/Upper respiratory tract infection	MF	(8, 42, 43, 48, 54–57)
Influenza	MF	(42)
Tuberculosis	MF PV	(42, 49, 58–61) (61)
Hepatitis B reactivation	MF PV	(59, 62, 63) (62, 64)
*Pneumocystis jirovecii* pneumonia	MF PV	(62) (62)

MPN, myeloproliferative neoplasm; MF, myelofibrosis; PV, polycythemia vera.

### 3.1 Clinical Trials and Retrospective Series

#### 3.1.1 Myelofibrosis

Due to the availability of more long-term safety reports, most of the data on JAK inhibitors-associated infections in MPNs concern MF patients. In the COMFORT-I trial, at a median 3-year follow-up, the incidence of urinary tract and Herpes zoster infections, the most common ones during randomized treatment with ruxolitinib, were not increased by long-term therapy, being respectively 10.5% in 0–12 months, 6.7% in 12–24 months, 7.7% in 24–36 months, 6.0% after ≥36 months, and 2.1% in 0–12 months, 3.5% in 12–24 months, 3.4% in 24–36 months, and 0% after ≥36 months. No other opportunistic infections occurred with long-term ruxolitinib therapy (41). According to the final analysis at 5-year follow-up, while grade 1/2 Herpes zoster infections were recorded at higher rate in ruxolitinib-treated subjects, other infections, including pneumonia, sepsis, upper respiratory, and urinary tract infections, displayed similar rates between ruxolitinib- and placebo-treated patients (54). In the COMFORT-II trial, the only grade 3/4 infectious AE was pneumonia, 1% in the ruxolitinib group *vs.* 5% in the best available therapy (BAT) group, being all other infections of grade 1/2 (8). At 5-year follow-up the longer exposure to ruxolitinib did not determine a significant increase in incidence of infections (65). In the JUMP trial, a ruxolitinib single-arm expanded-access study enrolling 2.233 MF patients, all-grade infections included urinary tract infection (6.0%), pneumonia (5.3%), Herpes zoster (3.6%), influenza (3.0%), and oral Herpes (1.6%). Tuberculosis occurred in three patients and legionella pneumonia in one; no HBV reactivation was instead observed (42). The ROBUST phase II study evaluated safety and efficacy of ruxolitinib in 48 intermediate‐1, intermediate-2, and high‐risk MF patients. The most common infections were of the urinary tract (16.7%), lower (14.6%) and upper (10.4%) respiratory tract, and nasopharyngitis (6.3%), with no reports of Herpes zoster, HBV, or tuberculosis (43).

In a further real-life investigation, 507 MF patients, 128 treated with ruxolitinib and the remaining with cytoreductive agents, were retrospectively evaluated to investigate incidence and risk factors of infectious complications. Overall, 112 (22%) patients experienced 160 infectious events, most being bacterial (78%) and affecting mainly the respiratory tract. While the rate of infections was higher in ruxolitinib‐treated subjects (44 *vs.* 20%), attention was raised on the possible impact of confounding factors such as high IPSS risk category and splenomegaly, both being the main risk factors for infections and prevailing in the JAK inhibitor cohort (55). In another series of 446 MF subjects retrospectively investigated, after a median ruxolitinib exposure of 23.5 months, 28% of patients experienced 161 infectious events, involving the respiratory tract in 50% of cases. While viral (14.9%) and fungal (2.5%) infections were also observed, bacteria were the most frequent etiological agent (68.9%). Previous infections and high IPSS risk score still correlated with higher infectious risk whereas splenomegaly reduction was associated with a decreased risk of subsequent infectious complications (56). In 70 MF patients treated with ruxolitinib according to clinical practice, after a median time of 8 months from therapy start, 12 subjects experienced 17 grade ≥2 infections, mainly bacterial, including a life‐threatening tuberculosis (58).

Data on momelotinib-related infectious complications can be retrieved mainly from the phase III randomized SIMPLIFY-1 and SIMPLIFY-2 trials. In the SIMPLIFY-1 study, grade ≥3 infections occurred in 7% of cases treated with momelotinib and in 3% of patients who received ruxolitinib (66). In the SIMPLIFY-2 trial, grade 3 urinary infection frequencies were 2% in the momelotinib and 0% in the BAT groups (44).

As far as fedratinib is concerned, in a double-blind, randomized phase III study enrolling 289 intermediate-2 or high-risk MF subjects assigned to fedratinib at a daily dose of 400 mg or 500 mg or to placebo, infections occurred in 42 and 39% of patients, respectively, in the 400 and 500 mg groups compared with 27% in the placebo group, the urinary tract infections being the most reported AE (45). In a phase II open-label randomized study on 31 MF patients treated with fedratinib, infections of any grade were recorded in 11 (35%) patients and of grade 3/4 in six (19%) subjects, the most frequent being of the urinary tract (46). Urinary tract infections, usually of grade 1/2, were the most common infectious complication (12 cases) also in the JAKARTA-2 trial, a single-arm phase II study assessing fedratinib in 97 MF patients (47).

In the PERSIST-1 trial enrolling 327 patients with higher-risk MF randomly assigned to pacritinib or BAT, the incidence of serious opportunistic infections in the pacritinib group was low: Herpes zoster infections were reported in 1% of patients, whereas pneumonia (three cases) was among the most frequent AEs leading to death (48). Also, in a further phase III clinical trial on 311 MF subjects randomized to pacritinib 400 mg once daily, pacritinib 200 mg twice daily, or BAT, pneumonia was a serious AE with 4, 7, and 3% of cases, respectively, in the pacritinib once daily, twice daily, and BAT groups (57).

#### 3.1.2 Polycythemia Vera

Data on ruxolitinib-associated infectious complications in PV are derived from the RESPONSE, RESPONSE-2, and RELIEF clinical trials (67). In the RESPONSE study addressed to HU-resistant or -intolerant PV patients with splenomegaly, the rate of any grade infections was 41.8% in the ruxolitinib group and 36.9% in the standard-therapy arm, while grade 3/4 infections were respectively 3.6 and 2.7%. Herpes zoster infections, all of grade 1/2, were recorded in seven (6.4%) patients in the ruxolitinib group as compared with no cases on standard therapy (9). At 80-week follow-up the rate of all infections was 29.4 per 100 patient-years of exposure in the ruxolitinib group, 27.8 in the cross-over cohort and 58.4 in the BAT arm. The rate of Herpes zoster infection was higher in patients originally randomized to ruxolitinib or treated with ruxolitinib after crossover than in BAT (50). The long-term ruxolitinib safety was then evaluated at 5 years. In the ruxolitinib arm, except Herpes zoster, which was more frequent in this group, the rate of infections was lower, 18.9 per 100 patient-years of exposure *vs.* 19.1 in the crossover population and 59.8 in the BAT cohort (51). The RESPONSE-2 study randomized to ruxolitinib or BAT PV patients resistant or intolerant to HU without splenomegaly. In the ruxolitinib group, grade 3/4 infections were reported in 3% of patients and grade 1/2 Herpes zoster infection in 1% of subjects while in the BAT cohort occurred respectively in 1% and in none of the cases (10). At week 80 the rates of all grade and grade 3/4 infections per 100 patient-years of exposure were 24.9 and 2.3, respectively, in the ruxolitinib arm *vs.* 33.7 and 3.7 in the BAT cohort. Frequency of Herpes zoster infections was higher in patients receiving ruxolitinib; in this group, however, no pneumonia or tuberculosis reactivation was reported (52). In the RELIEF trial, recruiting patients with an adequate hematocrit control on HU but still experiencing PV‐related symptoms randomized to ruxolitinib or HU, the ruxolitinib safety profile was similar with that reported in the RESPONSE and RESPONSE-2 studies. Infectious complications were generally grade 1/2, with Herpes zoster infection being observed in only one patient in the ruxolitinib arm (53).

#### 3.1.3 Unselected MPNs

In a retrospective study enrolling 202 cases treated with ruxolitinib and a control cohort of 73 ruxolitinib-naïve MPN subjects, infections usually of grade 1/2 occurred in 38.4% of ruxolitinib-naïve and 42.6% of ruxolitinib-treated patients, with upper respiratory infections, urinary tract infections, and pneumonia being the most frequent ones. Rate of Herpes zoster infection was 3.9 and 2.7%, respectively, in the ruxolitinib-treated and ruxolitinib-naïve groups. After propensity score weighting, there was no difference in risk of infection between the ruxolitinib-treated and ruxolitinib-naïve cohorts (68).

### 3.2 Reviews, Case Reports, and Registries Database

Being the JAK inhibitor with the longest time since approval, both exhaustive systematic reviews (27, 62) and numerous case reports have been published on ruxolitinib-associated infectious complications. Together with a review of the literature, Lussana et al. also performed a meta‐analysis of interventional phase III studies on MF and PV patients. In the PV trials RESPONSE, RESPONSE-2, and RELIEF, as well as in the pooled analysis of the extended COMFORT-I and COMFORT-II studies, ruxolitinib turned out to be associated with a statistically significant increased risk of Herpes zoster infection. In the 28 case reports collected in the same review, the most frequent ruxolitinib-associated infections were tuberculosis, followed by HBV reactivation and *Pneumocystis jirovecii* pneumonia (62).

In a literature and institutional records search on ruxolitinib-treated subjects, 32 cases of opportunistic infection mainly in MF patients were retrieved, the most common being tuberculosis followed by cryptococcal infection and HBV reactivation (59). The issue of ruxolitinib-associated Mycobacterial tuberculosis infections was also investigated in a retrospective pharmacovigilance review based on the FDA Adverse Events Reporting System. Between January 2011 and December 2018, out of 4.666 reports of typical Mycobacterial tuberculosis recorded in the database, 91 were due to ruxolitinib compared with 4.575 cases due to all other drugs with a significant odds ratio (OR) at 9.2. Also, for atypical mycobacterial infections, the OR for ruxolitinib compared to all other drugs was significant at 8.3, indicating that patients on ruxolitinib were at increased risk of developing mycobacterial infections (60). In a similar retrospective study based on the French Pharmacovigilance database, between August 2012 and August 2017, in 24 MPN subjects and two GVHD cases all treated with ruxolitinib, 30 cases of infections were reported: nine were bacterial, five mycobacterial, ten viral, four fungal, one protozoan, and one non-specified opportunistic infection, being Herpes zoster the most frequently identified pathogen (49). A retrospective study on 65 MF/PV patients treated with ruxolitinib between July 2011 and June 2018 recorded two mycobacterial infections (3% of patients), one due to Mycobacterial tuberculosis and one to Mycobacterium avium complex, a higher rate than reported in the original randomized studies (61). The several cases of tuberculosis reported in clinical trials as well as in registries database and in case reports indicate an increased risk of developing this infectious complication in ruxolitinib-treated subjects. Therefore, before starting ruxolitinib, a tuberculosis history and, in the presence of significant risk factors, a screening with Tuberculin Skin Test or preferably IFN-γ Release Assay should always be performed. During ruxolitinib treatment, a regular follow-up aimed at early diagnosis of tuberculosis is also advisable, followed by an appropriate therapy when required (69).

Also, the risk of HBV reactivation in ruxolitinib-treated MPN patients, ranging from asymptomatic virus replication to severe hepatitis, was highlighted in several case reports (63, 64, 70, 71). Screening procedures including HBsAg, anti-HBs, and anti-HBc, and HBV-DNA if anti-HBc is positive are recommended (72, 73). HBV-seropositive patients should be treated with antiviral drugs such as entecavir and tenofovir due to their low viral resistance rate. Patients negative for HbsAg, anti-HBs, and anti-HBc should instead be considered for immunization (69). Recommendations of the German Standing Committee on Vaccinations (STIKO) also suggest vaccinations against influenza, Herpes zoster, and *Streptococcus pneumoniae* for individuals beyond the age of 60 and *Neisseria meningitidis* for those with a pre-existing disorder of the immune system (74).

### 3.3 JAK Inhibitors and COVID-19

The management of the potential immunosuppressive effects and the relative risk of infectious complications during therapy with JAK inhibitors represent an even more pressing problem at a time like the current one dominated by the COVID-19 pandemic.

The SARS-CoV-2 coronavirus infection causing the coronavirus disease 2019 (COVID-19) is a highly contagious and life-threatening disease. The latter is critically associated with a high rate of respiratory failure, thrombo-hemorrhagic complications, and death, mainly due to an abnormal inflammatory response. Recent data have indeed highlighted a pivotal role for pulmonary immuno-thrombosis in the pathogenesis of severe COVID-19. SARS-CoV-2 enters airway epithelial cells *via* ACE2 receptors and subsequently triggers monocytes, macrophages, and T cells infiltration into the alveoli (75). This is then accompanied by local cytokine and chemokine generation, leading to elevated systemic levels of cytokines, including TNF-⍺, IL-1β, IL-6, and IL-8 (75).

Considering its pronounced anti-inflammatory properties, ruxolitinib was, therefore, hypothesized to be an effective therapy for COVID-19 (76). In a prospective study of 34 aged and high-risk comorbidity patients with severe COVID-19 infection, ruxolitinib was shown to be safe and associated with significant clinical improvement, especially in the lung function (77).

As MPN patients are prone to both thrombosis and bleeding, they call for special care during COVID-19. With this aim, the GIMEMA (Gruppo Italiano Malattie Ematologiche dell’Adulto) group conducted a survey of 34 Italian centers to study the prevalence of infections in this specific setting (78). A total of 1,095 patients were treated with ruxolitinib, 829 for MF (75.7%) and 266 for PV (24.3%), with 36 of them found positive for COVID-19: 13 (36%) were asymptomatic, 13 (36%) had flu-like symptoms, and 10 (27.8%) were affected by COVID-19-related pneumonia. Eight COVID-19-positive patients died with a death rate of 22%. As a result of this survey, it was found that the incidence of COVID-19 infection in MPN patients is rather low, and a certain protective function of ruxolitinib could not be ruled out.

A subsequent study by the European LeukemiaNet collected 175 MPN patients with COVID-19 during the first wave of the pandemic, from February to May 2020, in 38 international hematologic centers (79). Among the MPN phenotypes, patients with MF were the great majority (44%). Furthermore, they were at higher risk of mortality (48%) in comparison with both ET (25%) and PV (19%). When compared with the general COVID-19 population, the mortality ratio in this study was at least two to three times higher than the mortality rates reported by Johns Hopkins University in the same period and comparable to that reported in other hematologic malignancies (80–82). With regards to therapy ongoing at COVID-19 diagnosis, HU did not show significant correlations. In contrast, multivariable and propensity score matching analyses found an increased risk of death in patients who abruptly discontinued ruxolitinib treatment (79). Accordingly, JAK inhibitors should not be adjusted or discontinued in MPN patients to reduce the risk of COVID-19. On the contrary, stopping ruxolitinib in the event of COVID-19 infection may be harmful and should be avoided if clinically feasible. Otherwise, if ruxolitinib needs to be stopped, it should be tapered cautiously (27, 83).

Due to the immunomodulatory properties of ruxolitinib, the question arises whether response to SARS-CoV-2 vaccination might be impaired in MPN patients, in particular those under ruxolitinib therapy.

A recent study has already demonstrated that only a low proportion (17%) of solid organ transplant recipients mounted a positive antibody response to the first dose of SARS-CoV-2 mRNA vaccines, with those receiving anti–metabolite maintenance immunosuppression less likely to respond (84). In a subsequent study from the same authors, most of the patients had detectable antibody responses after the second dose, although participants without a response after dose 1 had generally low antibody levels. Consequently, a substantial proportion of transplant recipients likely remain at risk for COVID-19 after two doses of mRNA vaccine (85).

Focusing on hematological malignancies, different studies have already demonstrated a substantially reduced seroconversion rate post-COVID-19 vaccination in these patients, particularly in heavily treated patient groups, those with aggressive disease, marked cytopenias, and B-cell neoplasms (86–88).

On the contrary, patients with chronic myeloid neoplasms, including MPN, chronic myeloid leukemia, and myelodysplastic syndromes, seemed to show higher seroconversion rates than those reported in the former groups: more in details, reasonably high seroconversion rates following a single vaccine dose were observed in patients with CML and in MPN patients receiving interferon. However, humoral responses in certain MPN patients, especially in those receiving ruxolitinib, were found to be substantially impaired as compared to healthy adults of a similar age group (89–92). Even though the exact mechanism for this is not yet known, it could be suggested to be the result of both disease- and treatment-mediated immune dysfunction.

## 4 JAK Inhibitors’ Positive Effects on Inflammation in MPNs and Graft-*Versus*-Host Disease (GVHD)

The majority of MPN patients harbor mutations of the genes encoding for *JAK2*, *CALR*, or *MPL*, which result not only in the constitutive activation of the JAK-STAT signaling pathway but also of other pro-inflammatory signaling, in particular tumor necrosis factor (TNF)/nuclear factor k-light-chain-enhancer of activated B cells (NF-kB) pathways, in mutated hematopoietic stem cells and their progeny (93–95). *In vitro* studies have shown that the increased production of pro-inflammatory cytokines results from both an increase in the percentage of cytokine-secreting cells, as well as augmented cytokine secretion per *cell* (93). In addition to production of inflammatory cytokines by the MPN clone, immune dysregulation also results from paracrine/endocrine effects on non-clonal hematopoietic and stromal cells (96–99).

Consequently, MPN patients, particularly those with MF, exhibit both uncontrolled myeloproliferation and abnormally elevated levels of circulating pro-inflammatory cytokines causing disease-related systemic symptoms (100).

More precisely, plasma cytokine profile, especially in the setting of PMF or post-PV/ET myelofibrosis have already been shown to be significantly altered (101), and high levels of IL-8, IL-2 receptor, IL-12, and IL-15 were suggested as prognostic indicators of inferior survival and increased rate of leukemic transformation (102). Interestingly, it has also been shown that TNF-α, a pro-inflammatory cytokine, can facilitate clonal expansion of *JAK2*V617F-positive cells in MPNs (103). Besides their direct influence on the neoplastic clones, it is well known that cytokines can profoundly influence the bone marrow microenvironment, in MPNs as well as in other myeloid neoplasms.

In such a context, although potentially responsible for the immunosuppressive effect of ruxolitinib, its anti-JAK1 inhibitory action also leads to a reduction of pro-inflammatory cytokines, with a consequent improvement of symptoms, quality of life, and ultimately, bone marrow fibrosis (104, 105). The clinical response has already been shown to be independent of the *JAK2* mutational status, but it was linked to suppression of increased serum levels of pro-inflammatory cytokines such as IL-6 and TNF-α (37).

In addition, JAK inhibitors’ immunomodulatory properties may also be beneficial in other specific settings, as highlighted using ruxolitinib in GVHD (106–110). A recent multicenter retrospective analysis of ruxolitinib as salvage treatment in patients with steroid-resistant (SR) acute or chronic GVHD found an overall response rates (ORR) of >80% and 6-month survival rates ranging from 79% (acute disease) to 97% (chronic disease) (108). Based on the evidence supporting a role in preventing GVHD, ruxolitinib received in 2016 Breakthrough Therapy Designation from the US FDA for the treatment of GVHD (111).

In a more recent phase II study (REACH1) involving patients with grades II to IV SR acute GVHD, an overall response was achieved by 54.9% of the patients at day 28, including 26.8% with complete responses. Best ORR at any time was 73.2%, with a median duration of response of 345 days, thus producing durable responses and encouraging survival compared with historical data in patients with an otherwise dismal prognosis (112). In a subsequent randomized, open-label, phase III trial (REACH2) comparing the efficacy and safety of ruxolitinib with the investigator’s choice of therapy in the same patients’ setting, ORR at day 28 was higher in the ruxolitinib group than in the control group (62 *vs.* 39%; *p*<0.001), with a median overall survival of 11.1 *vs.* 6.5 months, respectively (113).

Accordingly, even though the treatment of acute GVHD has remained disappointing for decades, ruxolitinib and a few other agents seem to finally offer better therapeutic options, thus leading to a paradigm shift in the treatment of SR GVHD (114, 115).

## 5 Conclusions

The clinical use of JAK inhibitors in MPNs has led to a dramatic improvement of symptoms control, organ involvement, and quality of life. While JAK inhibitors are usually well tolerated, the issue of an increased risk of infections was raised by several clinical trials, retrospective series, and case reports (62). Indeed, JAK inhibitors may impair the immune response by mechanisms involving DCs, NKs, and CD4+ T cells as previously summarized. Even considering the intrinsic MPNs’ propensity to infectious complications, and other possible contributing factors such as previous treatments, concurrent immunosuppressive therapies, or comorbidities, available data suggest that incidence, and sometimes severity, of bacterial and viral infections in JAK inhibitors-treated MPN subjects are remarkable. Owing to the crucial therapeutic role of these drugs, their use must therefore be coupled with specific preventive measures (**Table 3**). Before JAK inhibitors therapy, screening for chronic HBV infection should be performed together with antiviral prophylaxis during treatment for suitable subjects. Monitoring of anti-HBc positive, HBsAg-negative patients is also indicated. Screening for latent tuberculosis is necessary, followed by therapy when needed. Antiviral and anti-pneumocystis prophylaxis could be considered in patients with specific risk factors (117). An adequate infections risk control is mandatory to fully exploit the JAK inhibitors’ therapeutic efficacy in MPNs.

**Table 3:** Proposal for the most frequent antiviral and antibiotic prophylaxis for ruxolitinib-treated MPN patients.

	Proposed prophylaxis	References
Herpes zoster	To evaluate a case-by-case basis (acyclovir)	(62)
Hepatitis B virus	Specialist referral (lamivudine *vs.* entecavir or tenofovir)	(116)
Tuberculosis	Specialist referral (isoniazid)	(117)

## Author Contributions

DC and AI wrote the paper and gave their final approval.

## Funding

The only funds used were those provided by the authors’ institutions.

## Conflict of Interest

The authors declare that the research was conducted in the absence of any commercial or financial relationships that could be construed as a potential conflict of interest.

## Publisher’s Note

All claims expressed in this article are solely those of the authors and do not necessarily represent those of their affiliated organizations, or those of the publisher, the editors and the reviewers. Any product that may be evaluated in this article, or claim that may be made by its manufacturer, is not guaranteed or endorsed by the publisher.
